# Nitric oxide donor sodium nitroprusside-induced transcriptional changes and hypocrellin biosynthesis of *Shiraia* sp. S9

**DOI:** 10.1186/s12934-021-01581-8

**Published:** 2021-04-28

**Authors:** Yan Jun Ma, Xin Ping Li, Yue Wang, Jian Wen Wang

**Affiliations:** 1 College of Pharmaceutical Sciences, Soochow University, Suzhou, 215123 China; 2 College of Life Sciences, Northwest Normal University, Lanzhou, 730000 China

**Keywords:** *Shiraia*, Sodium nitroprusside, Nitric oxide, Hypocrellin, Oxidative stress

## Abstract

**Background:**

Nitric oxide (NO) is a ubiquitous signaling mediator in various physiological processes. However, there are less reports concerning the effects of NO on fungal secondary metabolites. Hypocrellins are effective anticancer photodynamic therapy (PDT) agents from fungal perylenequinone pigments of *Shiraia*. NO donor sodium nitroprusside (SNP) was used as a chemical elicitor to promote hypocrellin biosynthesis in *Shiraia* mycelium cultures.

**Results:**

SNP application at 0.01–0.20 mM was found to stimulate significantly fungal production of perylenequinones including hypocrellin A (HA) and elsinochrome A (EA). SNP application could not only enhance HA content by 178.96% in mycelia, but also stimulate its efflux to the medium. After 4 days of SNP application at 0.02 mM, the highest total production (110.34 mg/L) of HA was achieved without any growth suppression. SNP released NO in mycelia and acted as a pro-oxidant, thereby up-regulating the gene expression and activity of reactive oxygen species (ROS) generating NADPH oxidase (NOX) and antioxidant enzymes, leading to the increased levels of superoxide anion (O_2_^−^) and hydrogen peroxide (H_2_O_2_). Gene ontology (GO) analysis revealed that SNP treatment could up-regulate biosynthetic genes for hypocrellins and activate the transporter protein major facilitator superfamily (*MFS*) for the exudation. Moreover, SNP treatment increased the proportion of total unsaturated fatty acids in the hypha membranes and enhanced membrane permeability. Our results indicated both cellular biosynthesis of HA and its secretion could contribute to HA production induced by SNP.

**Conclusions:**

The results of this study provide a valuable strategy for large-scale hypocrellin production and can facilitate further understanding and exploration of NO signaling in the biosynthesis of the important fungal metabolites.

**Supplementary Information:**

The online version contains supplementary material available at 10.1186/s12934-021-01581-8.

## Background

*Shiraia bambusicola* P. Hennings is a bambusicolous fungus parasitized on bamboo tender twigs and its fruiting bodies have been used in traditional Chinese medicine to stimulate blood circulation, relieve expectoration, cure rheumarthritis and relax muscle rigidity [1, 2]. Hypocrellins, main perylenequinone pigments isolated from the hypha and fruit-bodies of *Shiraia* fungi, have being developed as new non-porphyrin and reactive oxygen species (ROS)-generating photosensitizers in photodynamic therapy (PDT) for clinical application of anti-microbes, -cancers and -viruses [1, 3, 4]. Due to the difficulty of chemical synthesis of hypocrellins and the scarcity of wild fruiting bodies [5], *Shiraia* mycelium culture has become a promising production process of this new PDT agent [6]. More process strategies were applied to enhance hypocrellin production in the culture, including the condition optimization for the cultures [7], and application of biotic [8, 9] or abiotic elicitors [10, 11]. Nitric oxide (NO), a small molecular signal, was found to be involved in the induced hypocrellin production by a fungal elicitor from *Aspergillus niger* at 50 µg/mL [12] and a fungal elicitor PB90 at 5 nM [8]. The endogenous NO in *S. bambusicola* was also observed in extractive culture of *Shiraia* by Triton X-100 [13].

NO is an important regulatory molecule in mammals [14]. Recently, NO has been proved to be an essential signal in the elicitation of plant defense and secondary metabolite biosynthesis [15]. NO released by a NO donor sodium nitroprusside (SNP) could elicit plant secondary metabolites such as marjoram essential oil [16], total phenols and flavonoids in *Echinacea purpurea* roots [17], artemisinin in *Artemisia annua* hairy roots [18], and taxol in *Taxus yunnanensis* cells [19]. However, there are few reports on the effects of NO on fungal growth and secondary metabolism. NO may mediate L-arginine-induced conidiation of *Coniothyrium minitans* [20]. Wang and Higgins (2005) reported that the germination and development of *Colletotrichum coccodes* conidia were significantly inhibited by SNP at 100 µM [21]. A NO-releasing compound diethylenetriamine-NoNoate at 1.5 mM increased drastically the formation of cleistothecia in *A. nidulans*, suggesting a positive regulator of NO on fungal sexual development [22]. Zhao et al. demonstrated that NO was involved in the co-culture of *Inonotus obliquus* with *Phellinus morii* and triggered the biosynthetical pathway of phenylpropanoids, leading to an increased production of styrylpyrone derivatives [23]. SNP at 5 mM increased yield of ganoderic triterpenoid by 40.94% in submerged fermenting *Ganoderma lucidum* [24]. Although the endogenous NO generation was observed in *S. bambusicola* treated by fungal elicitors [8, 12] and Triton X-100 [13], the regulation roles of NO on fungal hypocrellin biosynthesis are still undetermined. Therefore, as a follow-up to our efforts on enhancing hypocrellin production [9, 25] and elucidating the role of NO on secondary metabolite biosynthesis [13, 26], we examined the effects of SNP on *Shiraia* hypocrellin biosynthesis. We also investigated the relationship between NO and elicitation responses including ROS generation, the activation of antioxidant defenses and hypocrellin production in *Shiraia* mycelium cultures. In this study, de novo transcriptome sequencing for *Shiraia* sp. S9 was performed for better understanding the NO regulation on hypocrellin biosynthesis. This study presented a novel elicitor of hypocrellin production and shed light on the relational roles of NO on the biosynthesis of secondary metabolites.

## Results

### SNP application released NO and enhanced hypocrellin production

**Fig. 1:**
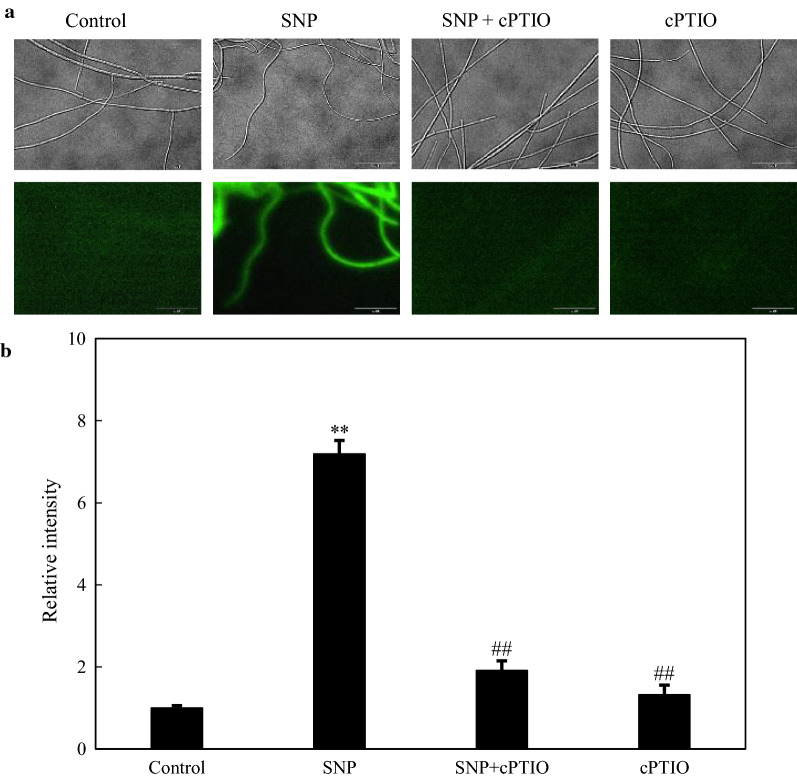
NO generation in SNP-treated mycelia of *Shiraia* sp. S9. **a** Bright-field image (above) and fluorescence microscopy of DAF-2-DA-stained mycelium (below) in cultures. SNP was added at 0.10 mM on day 3 of the culture. cPTIO (0.10 mM) was added 30 min prior to SNP treatment. The photos were taken after 2 h of SNP treatment. **b** NO accumulation (relative intensity of fluorescent ratio) in mycelium after SNP treatment. Values are mean ± SD from three independent experiments (**p < 0.01 *vs.* control, ^##^*p* < 0.01 *vs.* SNP group)

**Fig. 2:**
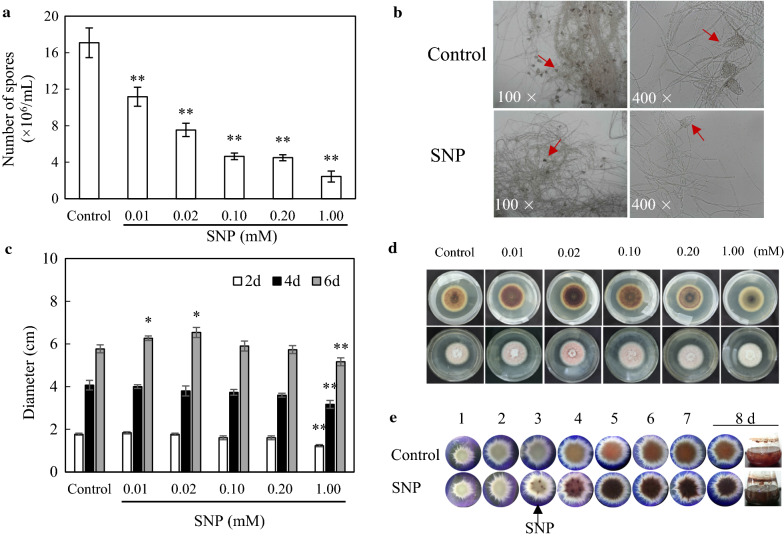
Effects of SNP on the growth and development of *Shiraia* sp. S9. **a** Effect of SNP (0.01–1.00 mM) on the generation of conidia of S9 strain on day 8 in solid medium culture. **b** The morphologic characteristics of S9 strain was kept on PDA with or without SNP (0.10 mM) treatment for 8 days. **c** The diameter of S9 colony treated by SNP at 0.01–1.00 mM for 2–6 days. **d** The colony morphology of S9 strain with SNP (0.01–1.00 mM) treatment for 6 days. **e** Pellet morphology (15 ×) in submerged culture of S9 strain under SNP treatment at 0.10 mM on day 3. The culture was maintained in 150-mL flask containing 50 mL of the liquid medium at 150 rpm and 28℃. The red *arrow* indicates pycnidium. The black *arrow* indicates addition time. Values are mean ± SD from three independent experiments (*p < 0.05 and **p < 0.01 *vs.* control)

After the application of SNP at 0.10 mM to 3-day-old cultures, we observed a rise of green fluorescence of NO indicator 4, 5-diaminofluorescein diacetate (DAF-2-DA) in *Shiraia* mycelia, whereas the relative fluorescent ration was markedly decreased by NO scavenger 2-(4-carboxyphenyl)-4, 4, 5, 5-tetramethylimidazoline-1-oxyl-3-oxide (cPTIO), indicating the release of NO in hyphae (Fig. 1a, b). On the solid medium, the concentration of fungal conidia was decreased from 17.08 × 10^6^ to 2.43 × 10^6^ spores/mL by SNP treatment (Fig. 2a). The pycnidium formation was suppressed by SNP (Fig. 2b). Although SNP at higher concentration (1.00 mM) inhibited the fungal growth, the diameter of fungal colony was increased on day 6 by SNP at 0.01 or 0.02 mM (Fig. 2c, d). In the liquid culture, there were not any notable changes in fungal biomass (Additional file 1: Figure S1A), pH value and sugar consumption (Additional file 1: Figure S2) in medium after SNP application at 0.10 mM. However, SNP decreased fungal pellet diameter by 16.09–25.73% (Additional file 1: Figure S1B) and the pellets were colored with dark red (Fig. 2e). The influence of SNP on hypocrellins including hypocrellin A (HA) and HC, and elsinochrome (A, B and C) in the culture was detected by HPLC (Additional file 1: Figure S3). Both the intracellular HA (1.22-fold of control) and EA (5.88-fold of control) were enhanced by SNP at 0.10 mM (Table 1). The extracellular HA was increased by 87.56%, while HC, EB and EC were not detected in cultural broth with or without SNP treatment.

**Table 1:** Effects of SNP on the individual hypocrellin production in submerged cultures of *Shiraia* sp. S9

PQ production (mg/L)	EC	EB	HC	EA	HA
Intracellular PQs in mycelia					
Control	0.54 ± 0.05	1.03 ± 0.13	5.68 ± 1.90	1.93 ± 0.04	47.05 ± 3.30
SNP	0.50 ± 0.03	1.29 ± 0.23	7.38 ± 0.29	11.34 ± 0.78**	57.54 ± 3.50*
Extracellular PQs in cultural broth					
Control	ND	ND	ND	0.13 ± 0.04	2.25 ± 0.20
SNP	ND	ND	ND	0.14 ± 0.02	4.22 ± 0.40**

The SNP at 0.10 mM was added into the mycelium cultures on day 3 and the culture was maintained in 150-mL flask containing 50 mL of the liquid medium at 150 rpm and 28 °C for 8 days. Values are mean ± SD from three independent experiments (*p < 0.05 and **p < 0.01 *vs.* control). ND indicates no detection

**Fig. 3:**
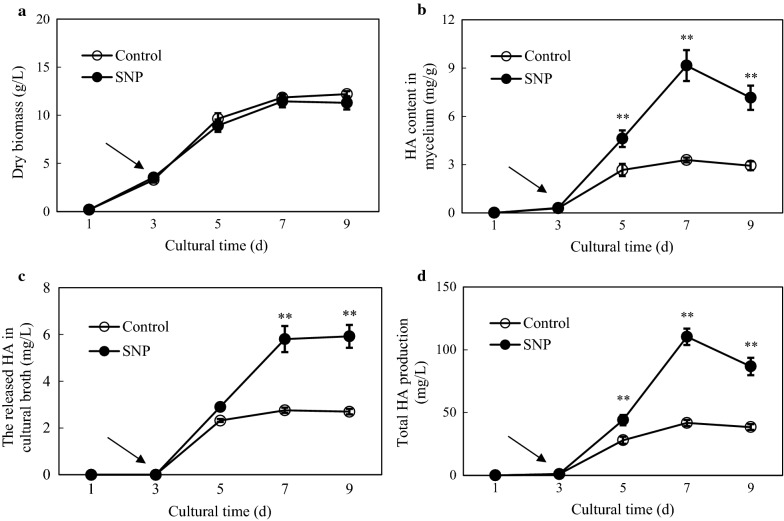
Time profiles of fungal biomass **a**, HA content in mycelia **b**, the released HA in cultural broth **c** and total HA production **d** in submerged culture of *Shiraia* sp. S9 under 0.02 mM SNP treatment on day 3. The culture was maintained in 150-mL flask containing 50 mL of the liquid medium at 150 rpm and 28 °C. The *arrow* represents the time of SNP addition. Values are mean ± SD from three independent experiments (**p < 0.01 *vs.* control)

As HA is a major bioactive hypocrellin constituent in *Shiraia* [9], we optimized the conditions (concentration and addition time) of SNP application to obtain higher HA production. SNP at lower concentrations (0.01–0.20 mM) had no obvious impacts on fungal biomass (Additional file 1: Figure S4A), but increased HA contents in mycelium and in cultural broth. The higher HA in mycelium (9.72 mg/g DW) and in cultural broth (4.64 mg/L) were obtained at 0.02 mM and 0.20 mM, respectively (Additional file 1: Figure S4B, C). When SNP at 0.02 mM was applied on different days, the higher contents of intracellular HA (9.97 mg/g DW) and the released HA in cultural broth (4.42 mg/L) were achieved on day 3 (Additional file 1: Figure S5). Hence, SNP application at 0.02 mM on day 3 of the culture was then used for subsequent culture. The time course of the effect of SNP on HA production in 9-day-old cultures was shown in Fig. 3. Under this optimal condition, SNP application did not suppress mycelial growth (Fig. 3a), but promoted the intracellular HA contents by 73.31–178.96% (Fig. 3b) and extracellular HA accumulation by 26.09–119.26% (Fig. 3c). The total HA production was enhanced to 110.34 mg/L on day 7, a 2.65-fold increase over the control without SNP addition (Fig. 3d).

### SNP-induced transcriptional changes of genes for hypocrellin biosynthesis

To examine the transcriptional changes of *Shiraia* sp. S9 after SNP treatment, RNA-Seq experiment was subsequently performed. There were altogether 84,275 unigenes assembled with an average length of 1,011.81 bp (base pairs) and an N50 of 5,399 bp (Additional file 1: Table S1, Figure S6). 98.62%, 54.40%, 37.37%, 30.86% and 90.40% of the total unigenes were resemble to known genes reported in the current databases in Additional file 1: Table S2. A total of 571 differentially expressed genes (DEGs) were identified (Additional file 2: Table S3), including up-regulated unigenes (355, 62.17%) and down-regulated unigenes (216, 37.83%) under SNP treatment (Additional file 1: Figure S7). These DEGs were categorized into the independent classification group “biological process (BP)”, “molecular function (MF)” and “cellular component (CC)” (Additional file 2: Table S4). Compared with the annotated unigenes of BP (270 DEGs) and CC (169 DEGs) (Additional file 1: Figure S8A, B), more than 322 DEGs were categorized into MF group (Additional file 1: Figure S8C). Within the MF category, DEGs (129 unigenes) assigned to ‘catalytic activity’ (GO:0003824) were of the highest proportion and other DEGs were mainly involved in ‘oxidoreductase activity’ (GO:0016491), ‘transporter activity’ (GO:0005215) and ‘transmembrane transporter activity’ (GO:0022857) items.

**Fig. 4:**
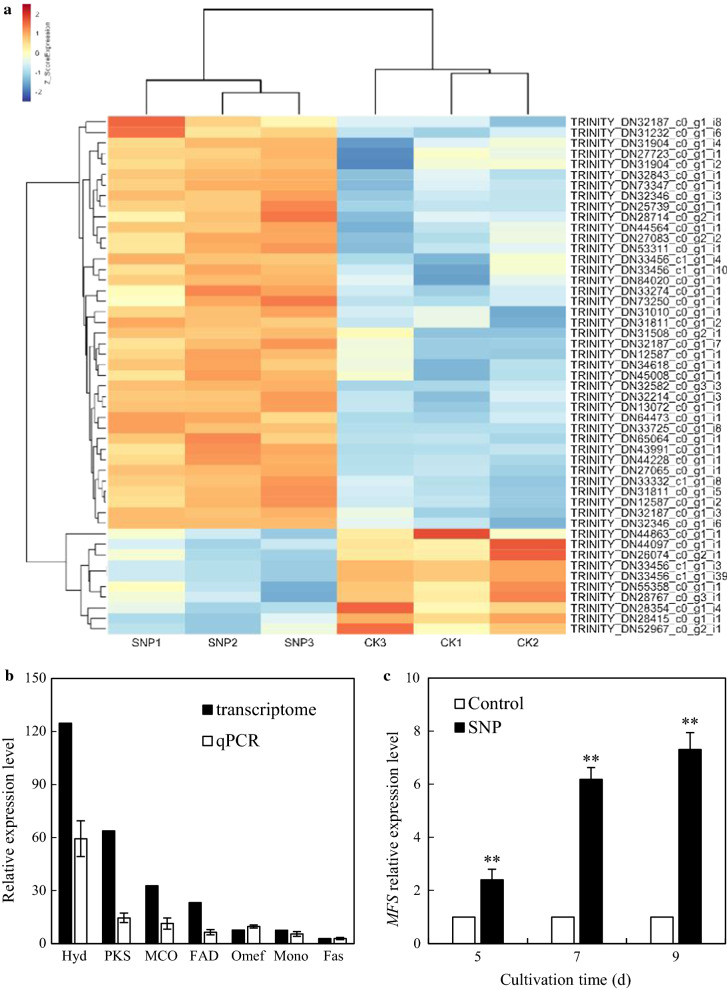
**a** Heat map of DEGs involved in hypocrellin biosynthesis of *Shiraia* sp. S9 by SNP. **b** Validation of the expression levels of unigenes related to hypocrellin biosynthesis of S9 strain by qRT-PCR on day 8. *Hyd*, TRINITY_DN84020_c0_g1_i1. *PKS*, TRINITY_DN73347_c0_g1_i1. *MCO*, TRINITY_DN31904_c0_g1_i4. *FAD*, TRINITY_DN33725_c0_g1_i8. *Omef*, TRINITY_DN28714_c0_g2_i1. *Mono*, TRINITY_DN33456_c1_g1_i4. *Fas*, TRINITY_DN43991_c0_g1_i1. **c** Validation of the expression levels of *MFS* (TRINITY_DN33647_c1_g1_i8) by qRT-PCR. The SNP treatment was the same as specified in Fig. 3. Values are mean ± SD from three independent experiments (**p < 0.01 *vs.* control)

Based on the reported gene clusters for hypocrellin biosynthesis [27–29], we explored the expression changes of DEGs associated with hypocrellin biosynthesis under SNP treatment. De novo sequencing and comparative analysis revealed 113 putative DEGs were enriched in 10 items related to fungal hypocrellin production (Additional file 2: Table S5 and S6), including ‘polyketide synthase’, ‘hydroxylase’, ‘probable metabolite transport protein’, and so forth. Among them, more than 75% DEGs which are associated with intracellular hypocrellin biosynthesis were up-regulated compared with control group (Additional file 2: Table S5 and Fig. 4a), such as laccase-like multicopper oxidase (*MCO*, TRINITY_DN31904_c0_g1_i4), conidial yellow pigment biosynthesis polyketide synthase (*PKS*, TRINITY_DN73250_c0_g1_i1), hydroxyindole-*O*-methyltransferase (*Omef*, TRINITY_DN28714_c0_g2_i1), FAD dependent oxidoreductase (*FAD*, TRINITY_DN33725_c0_g1_i8), phenol hydroxylase (*Hyd*, TRINITY_DN84020_c0_g1_i1), cytochrome P450 CYP2 subfamily (*Mono*, TRINITY_DN31508_c0_g2_i1), fasciclin (*Fas*, TRINITY_DN43991_c0_g1_i1) (Table 2). The expression levels of seven randomly selected unigenes related to hypocrellin biosynthesis were confirmed through qRT-PCR strictly (Fig. 4b), in which *Hyd* and *PKS* were more dramatically up-regulated by 59.35- and 14.59-fold, separately. The expression changes by qRT-PCR showed a similar tendency to those in transcriptome data (Table 2). In addition, it was found that more than 60% DEGs bound up with extracellular HA exportation were also up-regulated (Additional file 2: Table S6), such as major facilitator superfamily (*MFS*, TRINITY_DN33647_c1_g1_i8), ABC superfamily (*ABC*, TRINITY_DN73211_c0_g2_i1), secondary metabolites biosynthesis, transport and catabolism (*MTP*, TRINITY_DN30127_c0_g1_i1) (Table 2). Meanwhile, the SNP-induced up-regulation of transcriptional expression of selected unnigene *MFS* (TRINITY_DN33647_c1_g1_i8) from 2.40- to 7.31-fold was confirmed by qRT-PCR (Fig. 4c).

**Table 2:** Examples of DEGs involved in the hypocrellin biosynthesis, transport and transcription factors (TFs) of *Shiraia* sp. S9 by SNP

Unigene ID	Up/down	Fold change^a^	Description
**Hypocrellin biosynthesis**			
1. Laccase-like multicopper oxidase (*MCO*)			
TRINITY_DN31904_c0_g1_i4	Up	32.8	Multicopper oxidases [KOG1263]
TRINITY_DN31904_c0_g1_i2	Up	21.18	Multicopper oxidases [KOG1263]
2. Polyketide synthase (*PKS*)			
TRINITY_DN73347_c0_g1_i1	Up	63.84	Iterative polyketide synthase CazM [A0A0K0MCJ4.1]
TRINITY_DN73250_c0_g1_i1	Up	7.18	Conidial yellow pigment biosynthesis polyketide synthase [Q03149.2]
3. *O*-methyltransferase (*Omef*)			
TRINITY_DN28714_c0_g2_i1	Up	7.74	Hydroxyindole-*O*-methyltransferase and related SAM-dependent methyltransferases [KOG3178]
TRINITY_DN32187_c0_g1_i3	Up	4.49	Hydroxyindole-*O*-methyltransferase and related SAM-dependent methyltransferases [KOG3178]
4. FAD/FMN-dependent oxidoreductase (*FAD*)			
TRINITY_DN33725_c0_g1_i8	Up	23.27	FAD dependent oxidoreductase [OAL49443.1]
TRINITY_DN64473_c0_g1_i1	Up	4.7	Uncharacterized FAD-linked oxidoreductase ARB_02478 [D4AS41.2]
5. Hydroxylase (*Hyd*)			
TRINITY_DN84020_c0_g1_i1	Up	124.71	Phenol hydroxylase [KMK58601.1]
TRINITY_DN13072_c0_g1_i1	Up	18.1	Alkane hydroxylase 1 [Q9Y757.2]
6. Monooxygenase (*Mono*)			
TRINITY_DN25739_c0_g1_i1	Up	20.83	FAD-dependent monooxygenase andF [G3Y424.1]
TRINITY_DN31508_c0_g2_i1	Up	15.56	Cytochrome P450 CYP2 subfamily [KOG0156]
7. Fasciclin (*Fas*)			
TRINITY_DN43991_c0_g1_i1	Up	2.93	Fasciclin and related adhesion glycoproteins [KOG1437]
**Hypocrellin transport**			
1. Major facilitator superfamily (*MFS*)			
TRINITY_DN33647_c1_g1_i8	Up	43.12	Major facilitator superfamily [KOG0255]
TRINITY_DN6606_c0_g1_i1	Up	37.62	MFS quinate transporter-like protein QutD [OAL06848.1]
2. ATP-binding cassette transporter (*ABC*)			
TRINITY_DN73211_c0_g2_i1	Up	2.60	ABC superfamily [KOG0065]
TRINITY_DN30218_c0_g1_i2	Down	2.47	ABC superfamily [KOG0055]
3. Metabolite transport protein (*MTP*)			
TRINITY_DN30127_c0_g1_i1	Up	4.02	Secondary metabolites biosynthesis, transport and catabolism [KOG0222]
TRINITY_DN25695_c4_g2_i1	Up	2.23	Secondary metabolites biosynthesis, transport and catabolism [KOG1208]
**Transcription factors (TFs)**			
1. ERF			
TRINITY_DN31904_c0_g1_i4	Up	5.04	Hypothetical protein SNOG_06494 [XP_001796864.1]
TRINITY_DN31580_c0_g1_i1	Up	4.67	Hexose transporter-like protein [OAK96729.1]
2. C3H			
TRINITY_DN33456_c0_g1_i1	Up	2.36	P-loop containing nucleoside triphosphate hydrolase protein [OAG15388.1]
TRINITY_DN31301_c0_g1_i7	Up	2.02	ATP binding [KZM25621.1]
3. MYB			
TRINITY_DN33687_c0_g1_i33	Up	7.01	U4/U6-associated splicing factor PRP4 [KOG0670]
TRINITY_DN33611_c1_g2_i5	Up	6.67	ATP-dependent RNA helicase MSS116 [OAL02684.1]
4. NAC			
TRINITY_DN33640_c0_g1_i50	Down	8.87	Hypothetical protein IQ06DRAFT_327513 [OAK97976.1]
TRINITY_DN33640_c0_g1_i25	Down	8.48	Hypothetical protein SNOG_15601 [XP_001805746.1]
5. C2H2			
TRINITY_DN28764_c0_g1_i5	Down	2.74	Acetyl-CoA synthetase-like protein [OAL06367.1]
TRINITY_DN28413_c0_g1_i2	Down	2.61	Hypothetical protein SNOG_13514 [XP_001803722.1]
6. bHLH			
TRINITY_DN33276_c0_g1_i17	Down	7.94	Aldehyde dehydrogenase [OAK94834.1]
TRINITY_DN33276_c0_g1_i28	Down	6.32	Aldehyde dehydrogenase [OAK94834.1]

^a^Fold change, up: ratio (S2/S1); down: ratio (S1/S2). S1, the FPKM value of the unigene in control group; S2, the FPKM value of the unigene in SNP group

As shown in Additional file 2: Table S7, a total of 6440 unigenes were classified into 55 transcription factor (TF) groups, including C2H2, Trihelix, bZIP, bHLH, MYB related and so forth. Among all the TFs identified, bHLH (12.61%), ERF (7.47%), and MYB-related TF (7.08%) were of a higher proportion. Furthermore, there were altogether 189 DEGs enriched into 27 TFs differently expressed under SNP treatment (Additional file 2: Table S8), in which 24 TFs (110 DEGs) were up-regulated while 20 TFs (53 DEGs) were down-regulated in *Shiraia* mycelia. Among the up-regulated TFs, ERF ranked the highest (12 DEGs), followed by C3H (12 DEGs), and MYB (11 DEGs) (Table 2). Meanwhile, in the down-regulated TFs, NAC ranked the highest (8 DEGs), followed by C2H2 (6 DEGs), and bHLH (5 DEGs). Some prominent expression changes of unigenes associated with the high proportion of TFs were listed in Table 2, including several hypothetical proteins (TRINITY_DN31904_c0_g1_i4, TRINITY_DN33640_c0_g1_i50, TRINITY_DN33640_c0_g1_i25 and TRINITY_DN28413_c0_g1_i2), two aldehyde dehydrogenases (TRINITY_DN33276_c0_g1_i17 and TRINITY_DN33276_c0_g1_i28), hexose transporter-like protein (TRINITY_DN31580_c0_g1_i1), ATP binding (TRINITY_DN31301_c0_g1_i7).

### SNP-induced transcriptional changes of genes involved in membrane permeabilization

**Fig. 5:**
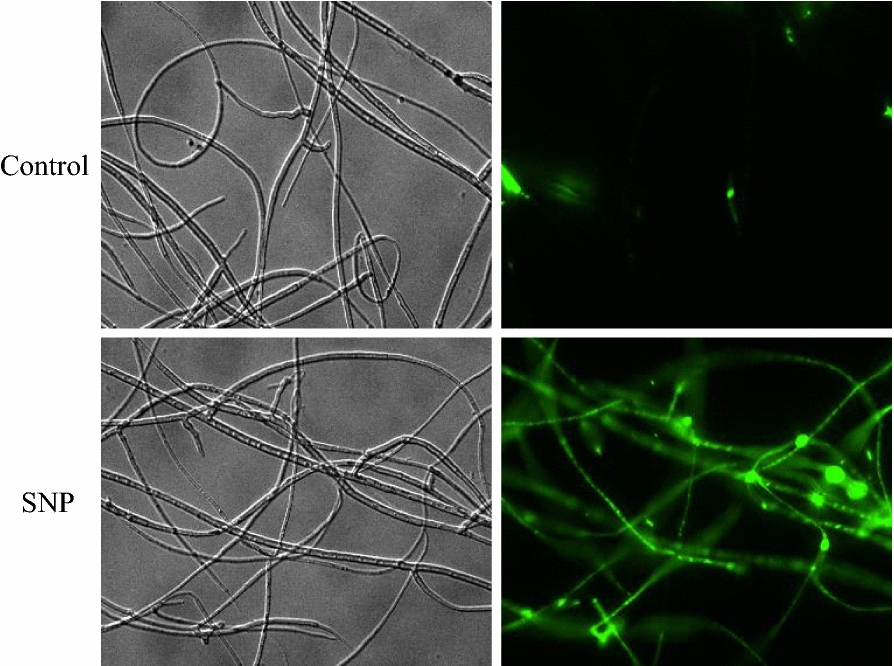
Effect of SNP treatment on membrane permeability in *Shiraia* sp. S9 mycelia (400 ×). The 8-day-old fungal mycelium was incubated with 0.50 μM SYTOX Green for 30 min. The SNP treatment was the same as specified in Fig. 3. Panels represent bright-field images on the left and green fluorescence images on the right

On the basis of the analysis results from GO classification (Additional file 2: Table S4), a large number of unigenes were enriched into ‘membrane’ (61 DEGs of GO:0016020), ‘transporter’ (51 DEGs of GO:0006810), ‘integral to membrane’ (36 DEGs of GO:0016021), ‘intrinsic to membrane’ (36 DEGs of GO:0031224), ‘membrane part’ (36 DEGs of GO:0,044,425), ‘transmembrane transport’ (34 DEGs of GO:0055085), ‘transporter activity’ (33 DEGs of GO:0005215) and ‘transmembrane transporter activity’ (30 DEGs of GO:0022857) items after SNP application. Hence, we explored the alteration of hyphal cell membrane permeability and membrane lipid components of *Shiraia* sp. S9. As shown in Fig. 5, the fluorescence was strengthened in SYTOX Green-stained cells after SNP treatment, indicating the increased permeabilization of cell membrane. Simultaneously, we found that the composition proportions of some saturated fatty acids such as palmitic (C16:0) and stearic (C18:0) were decreased by 19.08% and 23.97%, respectively (Table 3). On the contrary, the proportions of four unsaturated fatty acids including palmitoleic (C16:1), oleic (C18:1), erucic (C22:1) and nervonic (C24:1) were raised significantly, up to 6.23-fold (Table 3). Hence, the ratio of unsaturated fatty acids with saturated ones was enhanced to 0.57 in the hyphal cells of S9 strain under SNP treatment, a 4.07-fold of the control group, suggesting the enhancement of cell membrane fluidity.

**Table 3:** Effect of SNP treatment on fatty acid composition (% total fatty acid) of *Shiraia* sp. S9

Fatty acid composition	Total fatty acid (%)	
	Control	SNP
C16:0	24.21 ± 0.54	19.59 ± 1.07**
C18:0	15.02 ± 0.35	11.42 ± 0.16**
C23:0	0.18 ± 0.03	0.28 ± 0.05
C16:1	3.49 ± 0.24	8.28 ± 0.21**
C18:1	1.17 ± 0.10	4.79 ± 0.42**
C22:1	0.62 ± 0.04	3.86 ± 0.57**
C24:1	0.29 ± 0.03	0.82 ± 0.11**
Unsaturated/saturated fatty acid ratio	0.14 ± 0.01	0.57 ± 0.01**

The SNP treatment was the same as specified in Fig. 3. Ratio of unsaturated/saturated fatty acid = (C16:1 + C18:1 + C22:1 + C24:1)/(C16:0 + C18:0 + C23:0). Values are mean ± SD from three independent experiments. **p < 0.01 *vs.* control

### SNP-induced transcriptional changes of genes involved in oxidative stress

**Fig. 6:**
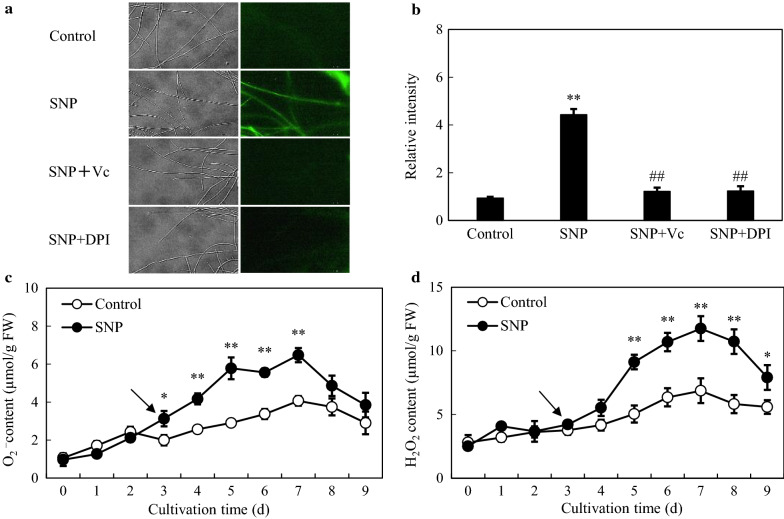
Effect of SNP treatment on ROS production in mycelium of *Shiraia* sp. S9. **a** Bright-field images (left) and fluorescence microscopy (right) of DCFH-DA-stained mycelia (400 ×). **b** ROS accumulation in mycelium after SNP treatment. Vc (0.01 mM) and DPI (5.00 μM) were added 30 min prior to SNP treatment. The contents of O_2_^−^ **c** and H_2_O_2_ **d** in mycelium of S9 by SNP. The photos and contents of ROS were taken and detected after 2 h of SNP treatment. The SNP treatment was the same as specified in Fig. 3. The *arrow* represents the time of SNP addition. Values are mean ± SD from three independent experiments (*p < 0.05, **p < 0.01 *vs*. control and ^##^*p* < 0.01 *vs*. SNP)

**Fig. 7:**
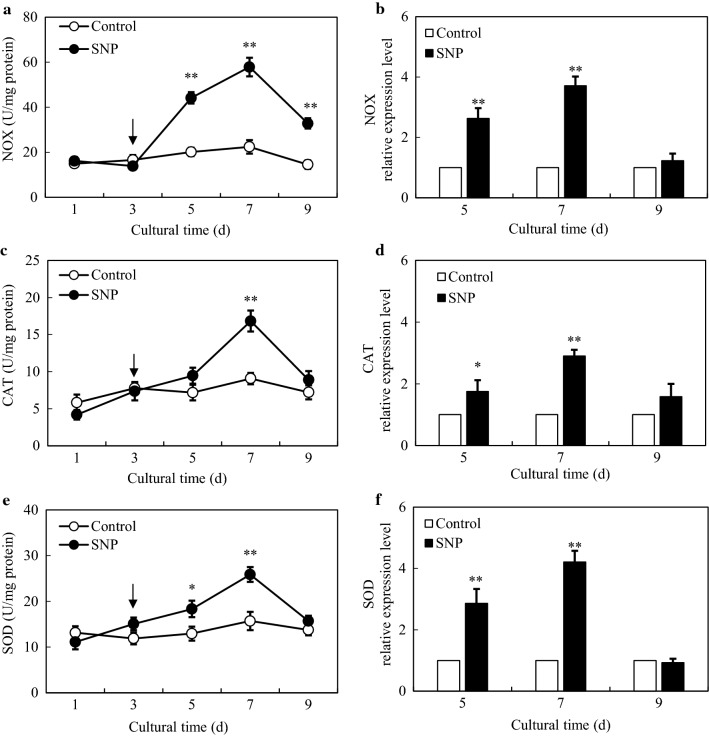
Effects of SNP treatment on activities and expressions of *NOX* (**a**, **b**), *CAT* (**c**, **d**) and *SOD* (**e**, **f**) in *Shiraia* sp. S9 mycelium on day 1–9. *NOX* (comp2367_c0_seq1), *CAT* (comp15524_c0_seq1), *SOD* (CL8477Contig1). The SNP treatment was the same as specified in Fig. 3. The *arrow* represents the time of SNP addition. Values are mean ± SD from three independent experiments (*p < 0.05 and **p < 0.01 *vs*. control)

According to the transcriptome analysis, there were 121 DEGs enriched into ‘oxidation–reduction process’ (GO:0055114), ‘oxidoreductase activity’ (GO:0016491), ‘electron carrier activity’ (GO:0009055) and ‘oxidoreductase activity, acting on paired donors, with incorporation or reduction of molecular oxygen’ (GO:0016705) items (Additional file 2: Table S4), suggesting an oxidative stress induced by SNP. By comparison between control and SNP groups (Fig. 6a), the green fluorescent signals of 2, 7-dichlorodihydroflurescein diacetate (DCFH-DA) in hyphal cells were brighter and more intense (4.72-fold, Fig. 6b), indicating the increase of ROS generation. When *Shiraia* sp. S9 was cultured in the presence of ROS scavenger vitamin C (Vc) and diphenyleneiodonium (DPI), an inhibitor of ROS generating enzyme NADPH oxidase (NOX) for 30 min prior to SNP application, the relative intensities of fluorescence in mycelia exhibited much more notable reduction of 72.39% and 72.14% (Fig. 6b) compared with the SNP group, separately. The content of superoxide anion (O_2_^−^) in mycelia was induced rapidly around 30 min of SNP treatment, reaching a highest value of 6.47 µmol/g FW (fresh weight) with time up to day 7, which was 59.14% higher than that of control (Fig. 6c). The change trends of hydrogen peroxide (H_2_O_2_) concentration were consistent with O_2_^−^ production. The generation of H_2_O_2_ was strikingly increased from 9.12 to 11.75 µmol/g FW after 2–4-day treatment of SNP and then decreased on day 7–9, but it was still higher than those of the control group (Fig. 6d). As shown in Fig. 7, the activities and expression levels of three oxidoreductases were significantly stimulated by SNP. The most significant stimulation of enzyme activities of NOX, catalase (CAT) and superoxide dismutase (SOD) occurred on day 7, which were 157.71%, 85.49% and 64.71% higher than those of control, respectively. Accordingly, the transcriptional expression levels of *NOX*, *CAT* and *SOD* were activated on day 5 or 7, and the strongest induction effect appeared on day 7, about 3.71-, 2.90- and 4.21-fold of control, separately.

## Discussion

SNP is a potent vasodilator used clinically to treat hypertensive emergencies and heart failure. In addition, it is widely used as nitric oxide donor in pharmacologic studies to investigate on the physiological roles of NO [14]. In plant cells or root cultures, SNP has been used as NO donor to elicit the production of important bioactive secondary metabolites such as anthocyanin and flavonol glycoside in *Pisum sativum* [30], and tropane alkaloids in hair roots culture of *Hyoscyamus reticulatus* [31]. More recently, the signal roles of NO in fungal developmental and metabolic biosynthesis have drawn more attention [32]. With the help of using NO donor SNP and NO scavengers, previous studies indicated that NO modulated the germination of *C. coccodes* conidia [21], hyphal elongation of *Magnaporthe oryzae* [33] and biosynthesis of fungal secondary metabolites such as styrylpyrone polyphenols, flavonoids and phenolics [23, 34]. However, it is less reported on SNP application as a chemical elicitor to enhance fungal metabolite production in mycelium cultures. In submerged cultures of *G. lucidum*, the eliciting effects of SNP at 0.5–5.0 mM on ganoderic triterpenoid production were investigated over 120 h [24]. The yields of ganoderic triterpenoids were increased by 40.94% at 72 h after SNP treatment at 5 mM. In our present study, NO elicited the biosynthesis of hypocrellins in *Shiraia* sp. S9, especially increased EA and HA contents in the mycelia (Table 1 and Additional file 1: Figure S3). After the optimization of the conditions for SNP application (the concentration and adding time) (Additional file 1: Figure S4 and S5), a higher production of HA (110.34 mg/L) was induced by SNP at 0.02 mM on day 7, a 2.65-fold increase over the control (Fig. 3). Compared with other elicitors used to promote HA production in *Shiraia* cultures including light irradiation [35, 36], ultrasound exposure [25] or microbial elicitors [9, 37], the chemical elicitor SNP has the advantages of lower cost, easy preparation, no growth suppression, and high efficiency for eliciting metabolite production in the mycelium cultures.

In our study, a rapid generation of endogenous NO was observed when SNP was applied (Fig. 1). The generated NO in mycelia was fully suppressed by a NO scavenger cPTIO, indicating exogenous SNP as a NO producer in the culture. Accumulating evidence showed NO could collaborate with the accompanied generation of ROS to form nitro-oxidative stress involved in human or plant diseases [14, 38]. Zheng et al. (2010) reported that the SNP (50 µM) promoted the H_2_O_2_ content in the hairy roots of *A. annua* elicited by fungal oligosaccharides [39]. SNP at 250 µM could enhance H_2_O_2_ level in adventitious roots of *E. purpurea* [17]. In this study, SNP treatment also resulted in an accumulation of ROS including O_2_^−^ and H_2_O_2_ in the mycelia (Fig. 6). Simultaneously, GO analysis demonstrated the enriched DEGs of ‘oxidation–reduction process’ (GO:0055114), ‘oxidoreductase activity’ (GO:0016491) and ‘electron carrier activity’ (GO:0009055) in the group of molecular function (Additional file 1: Figure S8 and Additional file 2: Table S4). Both the gene expressions of ROS generating NOX and the activities of antioxidant enzymes such as CAT and SOD were validated in SNP-treated mycelia (Fig. 7), suggesting that SNP could activate NOX for the increased oxidative stress in the mycelium cultures. In fungal cultures, ROS could provoke the production of fungal secondary metabolites, such as carotenoid in *Neurospora crassa* [40], deoxynivalenol in *Fusarium graminearum* [41], ochratoxin A in *A. ochraceus* [42]. In our previous researches, ROS generation and oxidative stress were reported as early signal-response events leading to HA biosynthesis of *Shiraia* under various abiotic elicitors, including the applications of surfactant Triton X-100 [10], lower intensity ultrasound [25] and light–dark shift [35]. These studies indicated the oxidative stress was involved in the hypocrellin biosynthesis of *Shiraia*. In our present study, we found 54 putative unigenes from 571 DEGs probably involved in hypocrellin biosynthesis after SNP treatment (Additional file 2: Table S5 and Fig. 4a). It was supposed that hypocrellins were biosynthesized in fungal cells via repetitive decarboxylative condensation of acetyl- and malonyl-CoA by polyketide synthase (*PKS*), methylation, decarboxylation and reduction by *O*-methyltransferase (*Omef*), monooxygenase/hydroxylase (*Mono*/*Hyd*) and multicopper oxidase (*MCO*), and then the core structure formation via the cooperation of fasciclin (*Fas*), laccase and berberine bridge enzyme (*BBE*) [28, 43, 44]. The deletion and overexpression of *SbaPKS* indicated its essential role in the hypocrellin biosynthesis of *Shiraia* sp. SUPER-H168 [45]. *SbaPKS* also had a moderating effect on the transcriptional expression of its adjacent genes *FAD*, *Omef*, *Mono* and *MCO* in the gene cluster. Overexpression of *Hyd* gene in *S. bambusicola* S4201 resulted in ultra-higher production of HA (1180.1 mg/L) [46]. Most of above-mentioned unigenes have been up-regulated significantly in our present experiment after SNP application, especially the expression of *Hyd* (59.35-fold) and *PKS* (14.59-fold) genes (Fig. 4b). The first transcription factor involved in HA biosynthesis is the zinc finger transcription factor zftf [29]. The *zftf* gene with a GAL4-like Zn(II)2Cys6 (or C6 zinc) binuclear cluster DNA-binding domain is located in the hypocrellin gene cluster. However, *zftf* is not the differentially expressed gene induced by SNP treatment (Additional file 2: Table S8). The up-regulated TFs were detected in the present study, including ERF, C3H and MYB (Table 2). These TFs have been studied extensively in initiating plant stress responses through involvement in the biosynthesis of secondary metabolites such as terpenoid indole alkaloids, taxol, flavonoids and proanthocynanins [47]. The challenge would be to elucidate the regulatory role of these TFs in regulation of HA biosynthesis. Taken together, the results from our present study have shown that SNP induced oxidative stress and activated genes and TFs for hypocrellin biosynthesis. The relationship between NO and ROS, and the mechanism of their mediation on hypocrellin biosynthesis are being further investigated.

It is worth noting that SNP could promote hypocrellin both inside the hyphae and from the medium (Fig. 3). In mycelium cultures after SNP application, the released HA in culture medium was increased by 26.09–119.26% (Fig. 3c). Although the increased extracellular HA accounts for only 5.26–6.83% of total HA production, the results still implicated the efflux of hypocrellins induced by SNP. Our GO classification revealed that the enriched DEGs were mainly in the categories of ‘membrane’ (GO:0016020), ‘integral to membrane’ (GO:0016021), ‘transmembrane transport’ (GO:0055085) and ‘transporter activity’ after SNP application (Additional file 1: Figure S8 and Additional file 2: Table S4). SNP treatment increased the proportion of total unsaturated fatty acids in the membranes of fungal hyphae (Table 3), leading to the enhanced membrane permeability (Fig. 5). On the other hand, membrane transporters have been reported to convey a photoactivated toxin cercosporin from *Cercospora* fungi for the auto-resistance [48]. Our RNA-Seq data also displayed that 32 unigenes encoding the transporter protein major facilitator superfamily (*MFS*) were up-regulated significantly (Additional file 2: Table S6). The qRT-PCR results also validated that the increases of gene expression levels of *MFS* (unigne No. TRINITY_DN33647_c1_g1_i8) (2.40- to 7.31-folds) after SNP application (Fig. 4b). These results indicated that SNP could induce changes of membrane permeability and activate membrane transporter such as *MFS* to facilitate the secretion of hypocrellins.

## Conclusion

Although endogenous NO generation was investigated in *Shiraia* mycelium cultures under the treatment of fungal elicitors [8, 12], the regulation on fungal metabolites by NO has not been well studied. To the best of our knowledge, it is the first time to use SNP as a chemical elicitor in mycelium cultures to promote hypocrellin productions. SNP at a higher concentration (0.02 mM) acted as a pro-oxidant, thereby raising the ROS generation, up-regulating biosynthetical genes for hypocrellin production. The increased membrane permeability and gene expression of member transporters induced by SNP was associated with efflux of hypocrellins. Our results showed the increase of total hypocrellin production is mainly attributed to the cellular hypocrellin biosynthesis and the efflux. When the mycelium cultures were treated by SNP at 0.02 mM for 4 days, the highest production (110.34 mg/L) of HA was achieved without any fungal growth depression. Although the mechanism of the SNP elicitation needs further investigation, the present study successfully provided a novel chemical elicitor for the biotechnological production of hypocrellins. This study is useful for hypocrellin production in a large-scale culture of *Shiraia* and facilitates the exploring of unknown genes and novel fungal secondary metabolites induced by SNP.

## Materials and methods

### Strain, media and culture conditions

The strain *Shiraia* sp. S9 (CGMCC16369) for hypocrellin production was obtained from fresh *Shiraia* fruit-body in our previous work [49]. The strain was maintained and stored at 4 °C on a potato dextrose agar (PDA) slant and initially grown at 28 °C for 8 days. The details of medium components and culture conditions were provided by our previous report [25]. The seed broth (2 mL) of S9 was transferred into a 150-mL Erlenmeyer flask containing 50-mL liquid medium and maintained in a rotary shaker at 150 rpm for 9 days at 28 °C.

### SNP application

SNP (Beyotime Biotechnology, Jiangsu, China) was dissolved in deionized water at 0.50 mM as a stock solution and filtered through a sieve (Ø0.22 μm) before use. To determine the optimal dosage of SNP for hypocrellin production, different dosages (0.01–1.00 mM) of SNP were added into the cultural broth on day 3. To confirm the effect of the addition time, 0.02 mM SNP was added into the mycelium culture on day 1–5 respectively.

### Measurement of medium pH, residual glucose and NO accumulation

The medium pH of the cultural broth was detected with pH Meter (FE20, Mettler Toledo, Zurich), and the residual glucose in cultural broth was measured using anthrone-sulfuric acid method [50]. To detect the NO accumulation in *Shiraia* sp. S9 mycelia, the NO-specific fluorescent probe DAF-2-DA (Sigma-Aldrich, St. Louis, MO, USA) was applied [51]. After 3 days of initial culture, DAF-2-DA (0.50 μM) was added to the cultural broth prior to SNP (0.10 mM) treatment. The NO scavenger cPTIO at 0.10 mM was added 30 min prior to SNP treatment [13]. After 2 h of incubation, a fluorescent microscope (Olympus Cell’R IX81, Center Valley, PA, USA) with excitation/emission wavelengths (470 nm/525 nm) was used for fluorescence observation.

### Observation of morphological characteristics

To analyze the effect of SNP (0.01–1.00 mM) on growth and morphological changes of *Shiraia* sp. S9 during the solid medium culture, the colony diameter was measured in triplicates (3 objects in a replicate). Photos were taken on day 6. To investigate the influence of SNP on fungal development, the 8-day-old colony was eluted with 10-mL deionized water. Subsequently, the fungal morphological characteristics were observed and photographed by a light microscope (CKX41, Olympus, Tokyo, Japan). To observe the influence of SNP on fungal growth during the submerged culture, SNP at 0.10 mM was added into the cultural broth on day 3. The mycelia were harvested and dried to constant weight at 60 °C to assess fungal biomass. The fungal pellets were viewed and photographed under a stereoscopic microscope (SMZ1000, Nikon, Tokyo, Japan). Pellet diameters were measured in triplicates (50 pellets per replicate).

### Analysis of cell membrane permeability

The membrane permeabilization of hyphal cell was detected by SYTOX Green dye, a high-affinity nucleic acid stain fluorescent probe (Eugene, Oregeon, USA) [52]. After 5 days of the initial culture of S9 strain, the harvested hyphae were incubated with the fluorescent probe solution (0.50 µM) for 30 min, and then the fluorescence intensity was observed and analyzed by means of an Olympus fluorescent microscope (CKX41, Tokyo, Japan) with the excitation/emission wavelengths at 488/538 nm respectively.

The components of mycelial fatty acids were extracted and analyzed via the protocols given in our previous report [10]. The detection of fungal fatty acids was carried out by using a gas chromatograph (Agilent7820, Palo Alto, CA, USA) with an Agilent DB-23 column (30.00 m × 0.25 mm dimension). The fatty acids were quantified with their internal standards purchased from Sigma-Aldrich (St. Louis, MO, USA).

### Measurement of ROS accumulation and activities of antioxidant enzymes

The ROS generation in hyphal cell was detected by DCFH-DA (Beyotime Biotechnology, Haimen, China) probe, which could penetrate the cell membrane freely [53]. After 2 h of SNP treatment, the harvested hyphae were incubated with the fluorescent dye solution (0.01 mM) for 1 h, and then the fluorescence intensity was observed and photographed by means of an Olympus fluorescent microscope (CKX41, Tokyo, Japan) with the excitation/emission wavelengths at 485/528 nm. The relative fluorescence value is defined to the ratio of fluorescence intensity at ‘SNP’ group to that at ‘Control’ group. Simultaneously, the contents of H_2_O_2_ and O_2_^−^ in the mycelia were assayed as previously described [54, 55]. The activities of NOX, CAT and SOD were measured using Enzyme Activity Assay Kit (Beyotime Biotechnology, Nanjing, China) according to the manufacturer’s protocol and previous reports [56–58].

To study the effects of ROS signal during the culture of S9 strain by SNP, Vc and DPI were used as ROS scavenger and NOX inhibitor, respectively. The concentrations of ROS scavenger and NOX inhibitor used in this work were chosen based on previous research [59]. Vc (0.01 mM) and DPI (5.00 µM) were added to the culture at 30 min prior to the application of SNP (0.02 mM) on day 3. The relative fluorescence value is defined to the ratio of fluorescence intensity at ‘SNP + Vc’ or ‘SNP + DPI’ groups to that at ‘SNP’ group.

### Detection of hypocrellin production

To analyze the influence of SNP on hypocrellin production in *Shiraia* sp. S9, SNP at 0.10 mM was added on day 3 and cultivated for 8 days. The hypocrellins in fungal mycelium and cultural broth were extracted based on the previous report [60]. The individual hypocrellin was quantified using a reverse-phase Agilent 1260 HPLC system (Agilent Co., Wilmington, USA) equipped with 250.0 mm × 4.6 mm Agilent HC-C18 column. Samples were eluted with a mobile phase of acetonitrile: water (65: 35, v/v) at 1 mL/min for 20 min and monitored at 465 nm. Individual hypocrellin was quantified separately with genuine standards provided by the Chinese National Compound Library (CNCL, Shanghai, China). Total hypocrellin production refers to the sum of intracellular and extracellular hypocrellin.

### Transcriptome sequencing, annotation and analysis

In this study, six samples with or without SNP treatment were obtained from three biological replicates (independent experiments) to establish cDNA libraries. Then, the HiSeq X Ten platform (Illumina, San Diego, CA, USA) was applied to sequence above cDNA libraries. The raw reads were cleaned and assembled according to our previous report [61]. All unigenes were subsequently annotated based on Basic Local Alignment Search Tool (BLAST) searches (version 2.2.31 +) against Gene Ontology (GO, http://www.geneontology.org/) database with a cut off *E* value of ≤ 1e^−5^. The raw RNA-seq data have been submitted to NCBI’s Gene Expression Omnibus (GEO) repository under accession number SRR7293200-7293205. In our study, we calculated the relative expression levels of genes via the fragments per kilobase per million reads (FPKM) method described by Mortazavi et al. [62]. Meanwhile, the significance of gene expression differences was evaluated using the cut-off criteria of |foldchange|≥ 2 and *p* value < 0.05.

### Quantitative real-time polymerase chain reaction (qRT-PCR)

Total RNA was extracted using RNAprep pure Plant Kit (Tiangen, Beijing, China) according to the instruction. The primer sequences of 18S ribosomal RNA (internal reference gene) and selected unigenes from NCBI database of SRR7293200-7,293,205 and PRJNA323638 were listed in Additional file 1: Table S9. The qRT-PCR was performed according to the method in our previous study [61]. And, the transcriptional expression levels of selected genes were calculated from cycle threshold values by using the 2^−△△CT^ method described in detail by Zhang et al. [63].

### Statistical analysis

Data analyses were carried out using Microsoft Excel and expressed as Mean ± Standard Deviation (SD). Student's *t*-test was applied for the comparison of the means between two groups. One-way analysis of variance (ANOVA) test was used for the comparison of the means among multiple groups. *p* < 0.05 is considered statistically significant.

## Supplementary Information

**Additional file 1: Table S1.** Illumina RNA-Seq reads and de novo assembly statistics of *Shiraia* sp. S9 by SNP. **Table S2.** Summary statistics of unigene annotation numbers of *Shiraia* sp. S9 by SNP. **Table S9.** Primers and relevant information of reference and target genes. F: forward primer, R: reverse primer. **Figure S1.** Effect of SNP at 0.10 mM on the growth and development of *Shiraia* sp. S9 in submerged culture. Time profiles of fungal biomass (A) and average pellet diameters (B) during the production culture. **Figure S2.** Time profiles of pH (A) and residual sugar (B) in *Shiraia* sp. S9 submerged cultures with the addition of SNP at 0.10 mM. **Figure S3.** Chromatograms of PQs in mycelium of *Shiraia* sp. S9 treated by SNP (0.10 mM) on day 3 and cultivated for 8 days. **Figure S4.** Effect of SNP concentration on (A) hyphal growth, (B) HA content in mycelium and (C) the released HA in cultural broth of *Shiraia* sp. S9. The strain was treated by SNP (0.01–1.00 mM) on day 3. **Figure S5.** Effect of SNP addition time on (A) hyphal growth, (B) HA content in mycelium and (C) the released HA in cultural broth of *Shiraia* sp. S9. SNP (0.02 mM) was added on different cultural time (1–5 d). **Figure S6.** The length distribution of unigenes of *Shiraia* sp. S9 by SNP. **Figure S7.** Heat map of DEGs summary of *Shiraia* sp. S9 by SNP. The up/down-regulated DEGs were detected by comparing RNA-Seq data of the unigenes of case group to control group (up: ratio > 1, down: ratio < 1). **Figure S8.** The cellular component (CC), biological process (BP) and molecular function (MF) of gene ontology (GO) categories of the DEGs in *Shiraia* sp. S9 under SNP treatment at 0.02 mM. The *arrow* represents the time of SNP addition. Values are mean ± SD from three independent experiments (**p < 0.01 *vs.* control). Different *letters* above the bars mean significant differences (*p* < 0.05).

**Additional file 2: Table S3.** Annotation of DEGs using NR, SWISS-Prot, KEGG, KOG and GO databases and their fold change with SNP treatment. **Table S4.** GO enrichment of *Shiraia* sp. S9 with SNP treatment. **Table S5.** Differentially expressed genes (DEGs) involved in hypocrellin biosynthesis of *Shiraia* sp. S9 under SNP treatment. **Table S6.** Differentially expressed genes (DEGs) involved in hypocrellin transport of *Shiraia* sp. S9 under SNP treatment. **Table S7.** Annotation of transcription factors in *Shiraia* sp. S9. **Table S8.** Differentially expressed genes (DEGs) involved in transcription factors of *Shiraia* sp. S9 under SNP treatment.

## Data Availability

All data generated or analyzed during this study are included in this published article and its Additional files.
