# Supplementary material for: Nitric oxide donor sodium nitroprusside-induced transcriptional changes and hypocrellin biosynthesis of Shiraia sp. S9
Source: Microb Cell Fact. 2021 Apr 28;20:92. doi: 10.1186/s12934-021-01581-8 (PMC8082767; doi:10.1186/s12934-021-01581-8)
Supplement: Supplementary file 1 — Additional file 1: Table S1. Illumina RNA-Seq reads and de novo assembly statistics of Shiraia sp. S9 by SNP. Table S2. Summary statistics of unigene annotation numbers of Shiraia sp. S9 by SNP. Table S9. Primers and relevant information of reference and target genes. F: forward primer, R: reverse primer. Figure S1. Effect of SNP at 0.10 mM on the growth and development of Shiraia sp. S9 in submerged culture. Time profiles of fungal biomass (A) and average pellet diameters (B) during the production culture. Figure S2. Time profiles of pH (A) and residual sugar (B) in Shiraia sp. S9 submerged cultures with the addition of SNP at 0.10 mM. Figure S3. Chromatograms of PQs in mycelium of Shiraia sp. S9 treated by SNP (0.10 mM) on day 3 and cultivated for 8 days. Figure S4. Effect of SNP concentration on (A) hyphal growth, (B) HA content in mycelium and (C) the released HA in cultural broth of Shiraia sp. S9. The strain was treated by SNP (0.01–1.00 mM) on day 3. Figure S5. Effect of SNP addition time on (A) hyphal growth, (B) HA content in mycelium and (C) the released HA in cultural broth of Shiraia sp. S9. SNP (0.02 mM) was added on different cultural time (1–5 d). Figure S6. The length distribution of unigenes of Shiraia sp. S9 by SNP. Figure S7. Heat map of DEGs summary of Shiraia sp. S9 by SNP. The up/down-regulated DEGs were detected by comparing RNA-Seq data of the unigenes of case group to control group (up: ratio > 1, down: ratio < 1). Figure S8. The cellular component (CC), biological process (BP) and molecular function (MF) of gene ontology (GO) categories of the DEGs in Shiraia sp. S9 under SNP treatment at 0.02 mM. The arrow represents the time of SNP addition. Values are mean ± SD from three independent experiments (**p < 0.01 vs. control). Different letters above the bars mean significant differences (p < 0.05). [file 12934_2021_1581_MOESM1_ESM.pdf]

Additional file 1

Nitric oxide donor sodium nitroprusside-induced transcriptional changes and hypocrellin biosynthesis of *Shiraia* sp. S9

Yan Jun Ma, Xin Ping Li, Yue Wang, Jian Wen Wang

✉ Jian Wen Wang
jwwang@suda.edu.cn; bcjwwang@gmail.com

Table S1. Illumina RNA-Seq reads and *de novo* assembly statistics of *Shiraia* sp. S9 by SNP.
Table S2. Summary statistics of unigene annotation numbers of *Shiraia* sp. S9 by SNP.
Table S9. Primers and relevant information of reference and target genes. F: forward primer, R: reverse primer.
Figure S1. Effect of SNP at 0.10 mM on the growth and development of *Shiraia* sp. S9 in submerged culture. Time profiles of fungal biomass (A) and average pellet diameters (B) during the production culture.
Figure S2. Time profiles of pH (A) and residual sugar (B) in *Shiraia* sp. S9 submerged cultures with the addition of SNP at 0.10 mM.
Figure S3. Chromatograms of PQs in mycelium of *Shiraia* sp. S9 treated by SNP (0.1 mM) on day 3 and cultivated for 8 days.
Figure S4. Effect of SNP concentration on (A) hyphal growth, (B) HA content in mycelium and (C) the released HA in cultural broth of *Shiraia* sp. S9. The strain was treated by SNP (0.01-1.00 mM) on day 3.
Figure S5. Effect of SNP addition time on (A) hyphal growth, (B) HA content in mycelium and (C) the released HA in cultural broth of *Shiraia* sp. S9. SNP (0.02 mM) was added on different cultural time (1-5 d).
Figure S6. The length distribution of unigenes of *Shiraia* sp. S9 by SNP.
Figure S7. Heat map of DEGs summary of *Shiraia* sp. S9 by SNP. The up/down-regulated DEGs were detected by comparing RNA-Seq data of the unigenes of case group to control group (up: ratio > 1, down: ratio < 1).
Figure S8. The cellular component (CC), biological process (BP) and molecular function (MF) of gene ontology (GO) categories of the DEGs in *Shiraia* sp. S9 under SNP treatment at 0.02 mM.
The *arrow* represents the time of SNP addition. Values are mean ± SD from three independent experiments (***p* < 0.01 *vs.* control). Different *letters* above the bars mean significant differences (*p* < 0.05).

Table S1. Illumina RNA-Seq reads and *de novo* assembly statistics of *Shiraia* sp. S9 by SNP.

**Attributes**	**Control**			**SNP**		
Samples	1	2	3	1	2	3
Total clean reads	51,601,366	47,683,624	44,648,930	47,180,148	48,394,762	51,812,366
Total unigenes (≥ 201 bp)	84,275					
Max length	33,762					
Min length	201					
Average length	1,011.81					
N50	5,399					

Table S2. Summary statistics of unigene annotation numbers of *Shiraia* sp. S9 by SNP.

**Annotation**	**Database**				
	NR [a]	Swiss-Prot [b]	KOG [c]	KEGG [d]	GO [e]
Numbers	46,278	25,530	17,537	14,481	42,421
Ratio (%)	98.62	54.40	37.37	30.86	90.40

a-NR, non-redundant, ftp://ftp.ncbi.nih.gov/blast/db.
b-Swiss-Prot, http://www.uniprot.org/downloads.
c-KOG, cluster of orthologous groups of proteins, ftp://ftp.ncbi.nih.gov/pub/COG/KOG/kyva.
d-KEGG, kyoto encyclopedia of genes and genomes, http://www.genome.jp/kegg/.
e-GO, gene ontology, http://www.geneontology.org/.

Table S9. Primers and relevant information of reference and target genes. F: forward primer, R: reverse primer.

**Genes name**	**Gene description**	**Sequence**
18S	Reference gene	F: ACGCAGCGAAATGCGATAAG<br>R: GGGCGAGAATCTTAGCAGCA
TRINITY_DN73347_c0_g1_i1	Iterative polyketide synthase CazM [A0A0K0MCJ4.1]	F: GCAGATACGCCCCTCACTAC<br>R: GTCGCTGGTAATATCGCCCA
TRINITY_DN43991_c0_g1_i1	Fasciclin and related adhesion glycoproteins [KOG1437]	F: TTTTACGATGCTACCCGCGA<br>R: CGAGAAAACCCGTTGATGCG
TRINITY_DN31904_c0_g1_i4	Multicopper oxidase [KOG1263]	F: TATGGCGCTTACGAGTGGAC<br>R: ACTCCCTGGCCGATAACGTA
TRINITY_DN28714_c0_g2_i1	Hydroxyindole-*O*-methyltransferase and related SAM-dependent methyltransferases [KOG3178]	F: TTGATGCGCATTTGACTGCC<br>R: ATCGACTCGGTGTTATGGGC
TRINITY_DN33725_c0_g1_i8	FAD dependent oxidoreductase [OAL49443.1]	F: TGTGACCGCCATCACCTTAC<br>R: TTCTCGTATGGGTGGGAAGC
TRINITY_DN84020_c0_g1_i1	Phenol hydroxylase [KMK58601.1]	F: TATGCTTACGGTTCCCCGTG<br>R: GGGCGAGAATCTTAGCAGCA
TRINITY_DN33456_c1_g1_i4	Cytochrome P450 monooxygenase yanC [G3Y416.1]	F: TCTCGGGGAATTATGGCACG<br>R: ACAACCGTTCTCGCATCAGT
TRINITY_DN33647_c1_g1_i8	Major facilitator superfamily [KOG0255]	F: TGGTCAGCGTTCTAGGCTTG<br>R: ACCCGTGTTCCACAATACCC
comp15524_c0_seq1	Catalase [GO:0046872]	F: CCGGGATCAAACCATGTGGA<br>R: GGTGGCTGGCAGGTAAATCT
CL8477Contig1	Superoxide dismutase [KOG0876]	F: GACGAAGGTGGAGAGTGCAA<br>R: TGCAGAGTATTCGGGTTCCG
comp2367_c0_seq1	Superoxide-generating NADPH oxidase heavy chain subunit A [KOG0039]	F: ATAACCGTTGACCGGCCATT<br>R: AAGATGAGTTTCCGCGGTGT

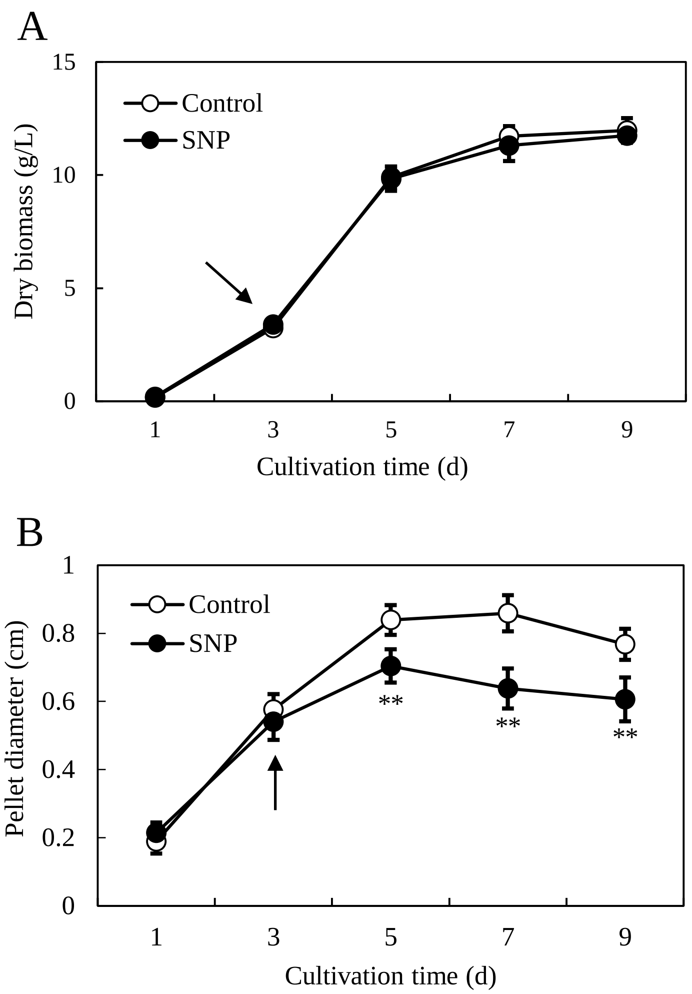

Figure S1. Effect of SNP at 0.10 mM on the growth and development of *Shiraia* sp. S9 in submerged culture. Time profiles of fungal biomass (A) and average pellet diameters (B) during the production culture.

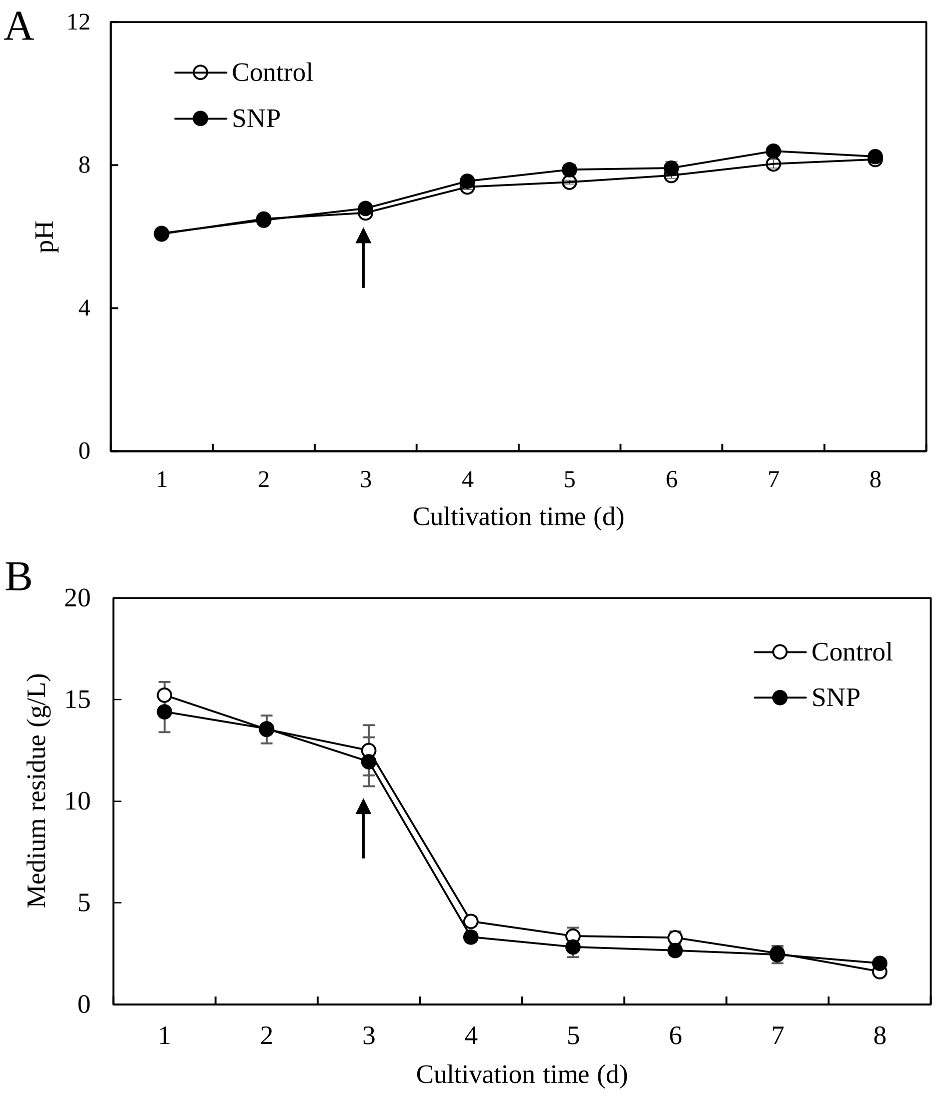

Figure S2. Time profiles of pH (A) and residual sugar (B) in *Shiraia* sp. S9 submerged cultures with the addition of SNP at 0.10 mM.

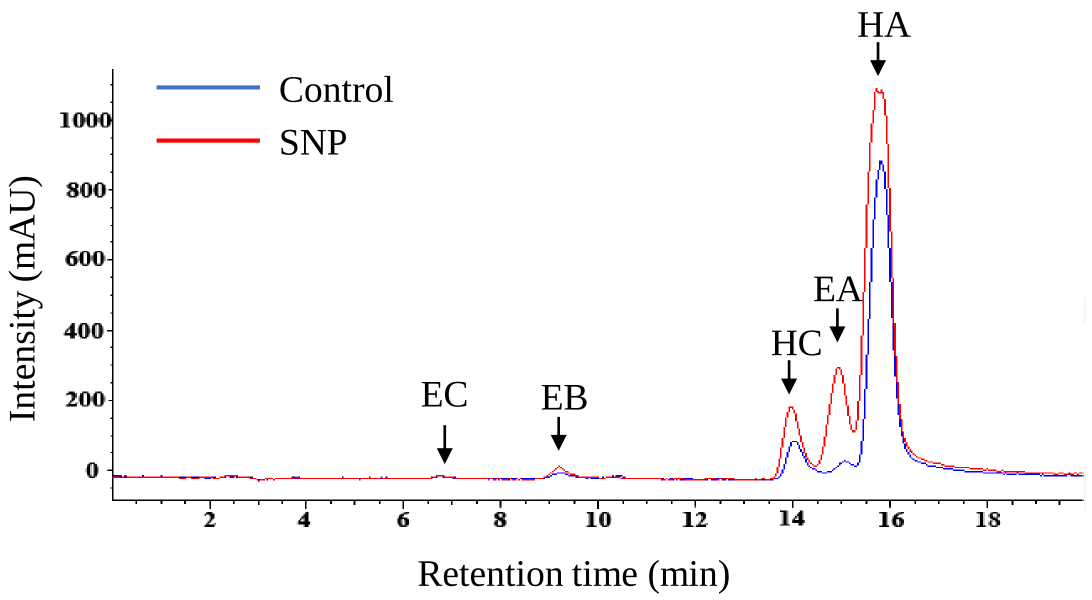

Figure S3. Chromatograms of PQs in mycelium of *Shiraia* sp. S9 treated by SNP (0.1 mM) on day 3 and cultivated for 8 days.

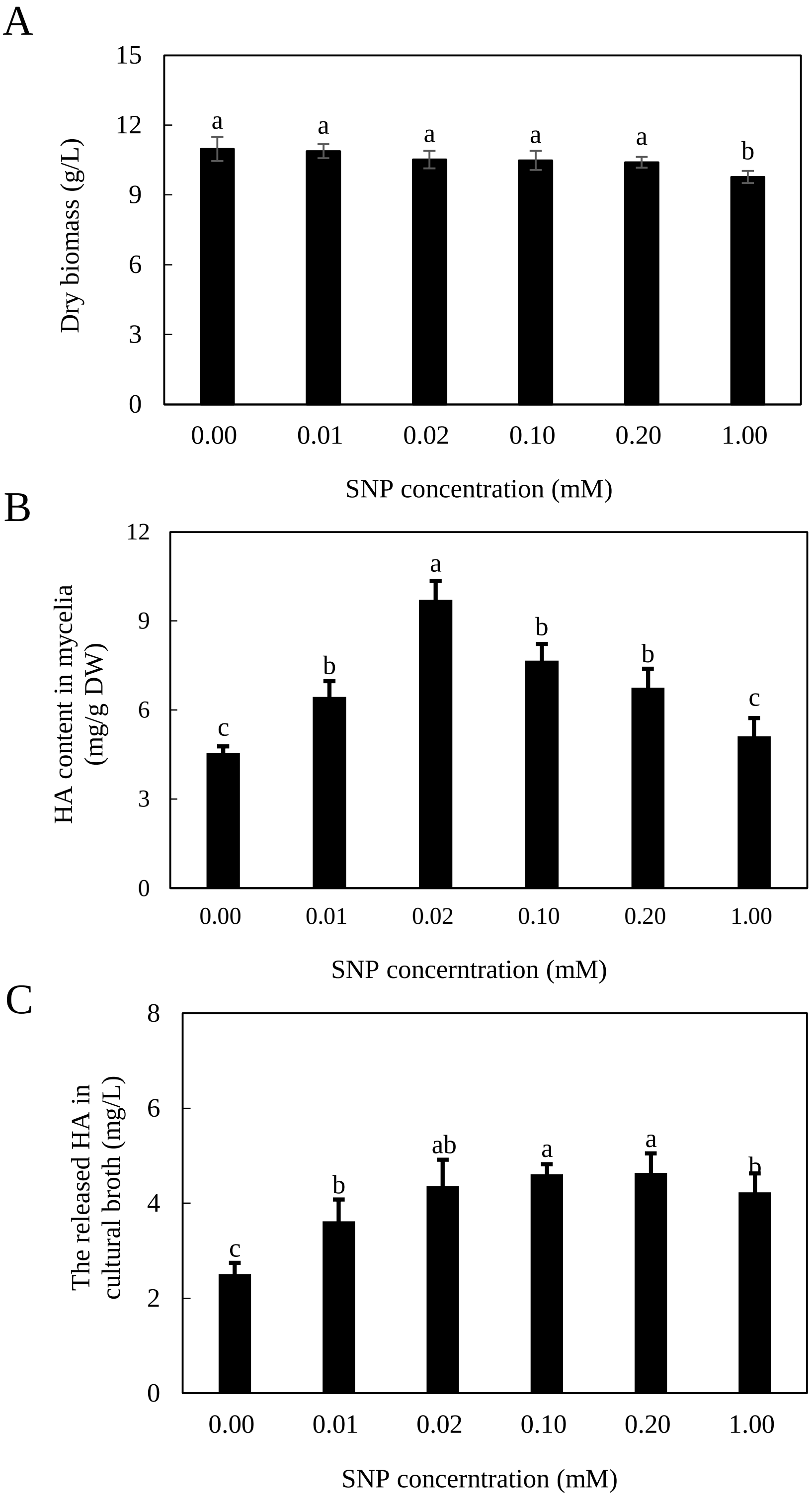

Figure S4. Effect of SNP concentration on (A) hyphal growth, (B) HA content in mycelium and (C) the released HA in cultural broth of *Shiraia* sp. S9. The strain was treated by SNP (0.01-1.00 mM) on day 3.

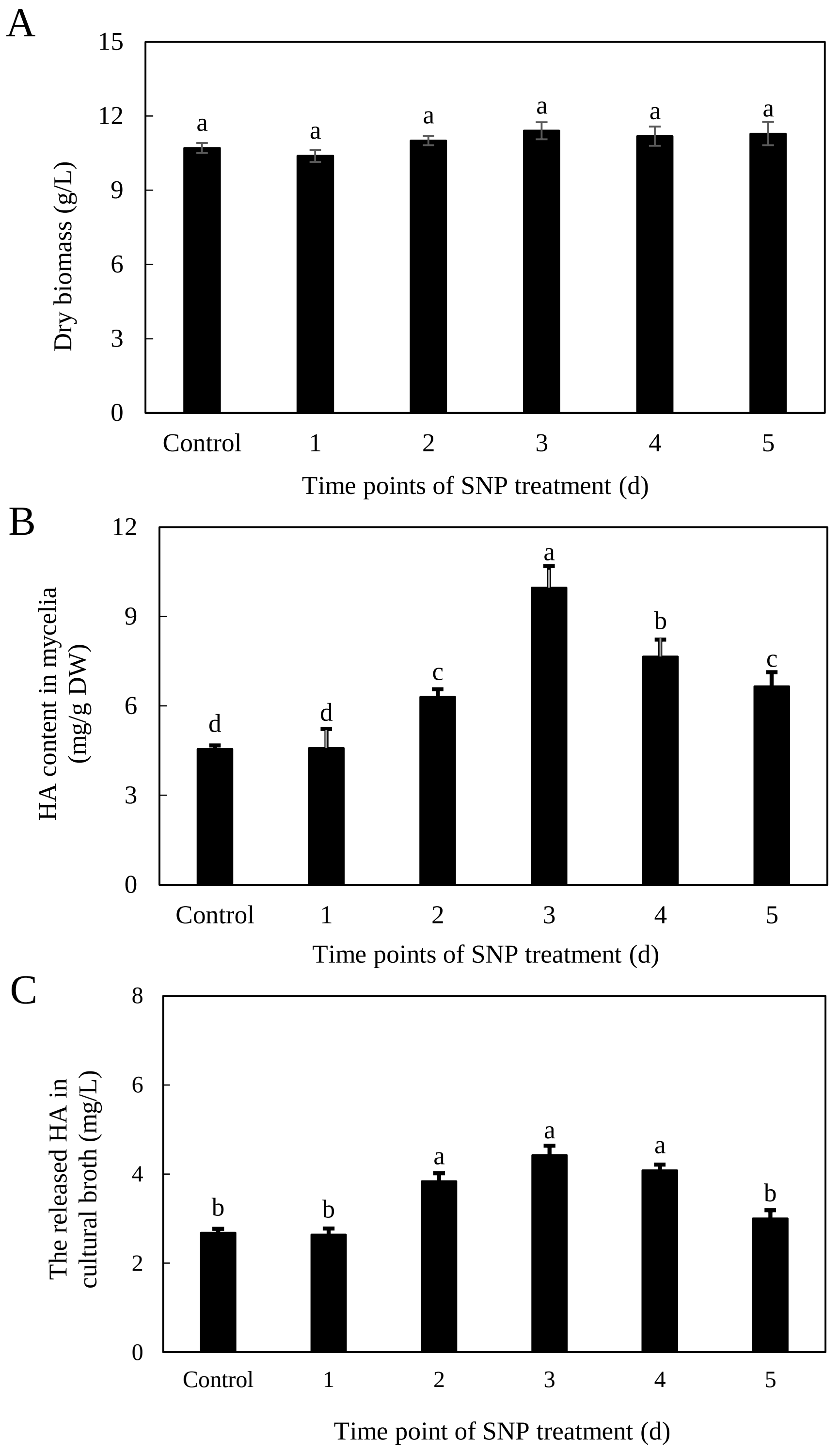

Figure S5. Effect of SNP addition time on (A) hyphal growth, (B) HA content in mycelium and (C) the released HA in cultural broth of *Shiraia* sp. S9. SNP (0.02 mM) was added on different cultural time (1-5 d).

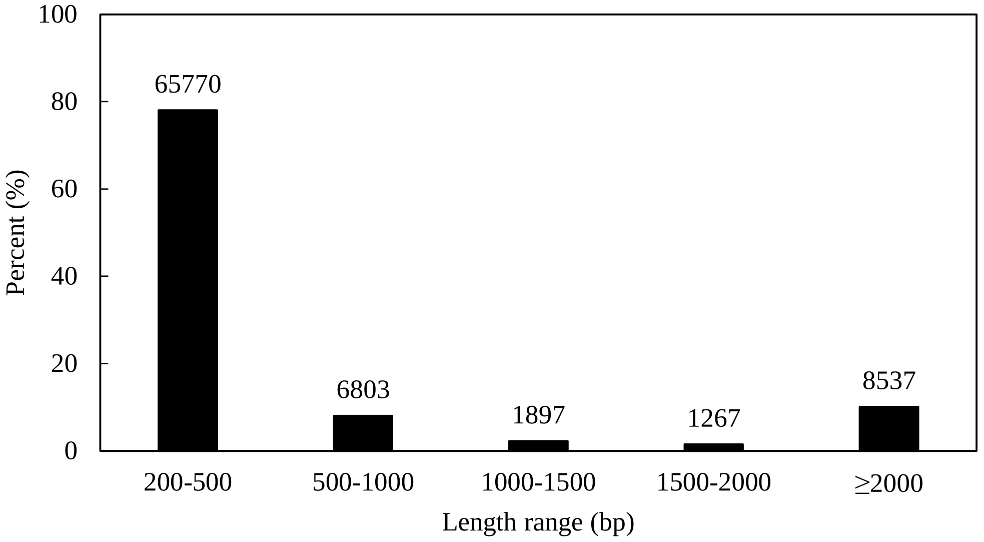

Figure S6. The length distribution of unigenes of *Shiraia* sp. S9 by SNP.

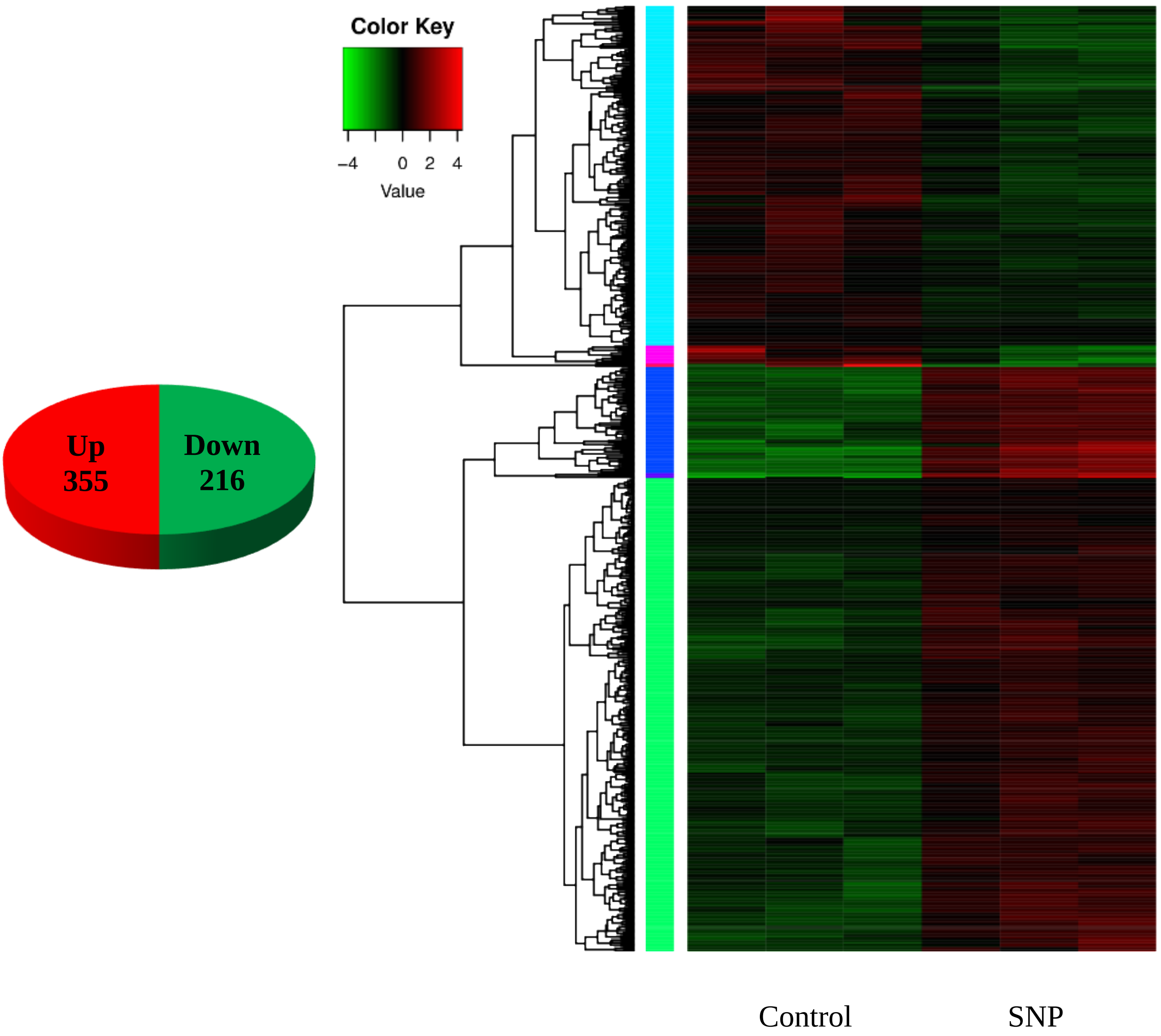

Figure S7. Heat map of DEGs summary of *Shiraia* sp. S9 by SNP. The up/down-regulated DEGs were detected by comparing RNA-Seq data of the unigenes of case group to control group (up: ratio > 1, down: ratio < 1).

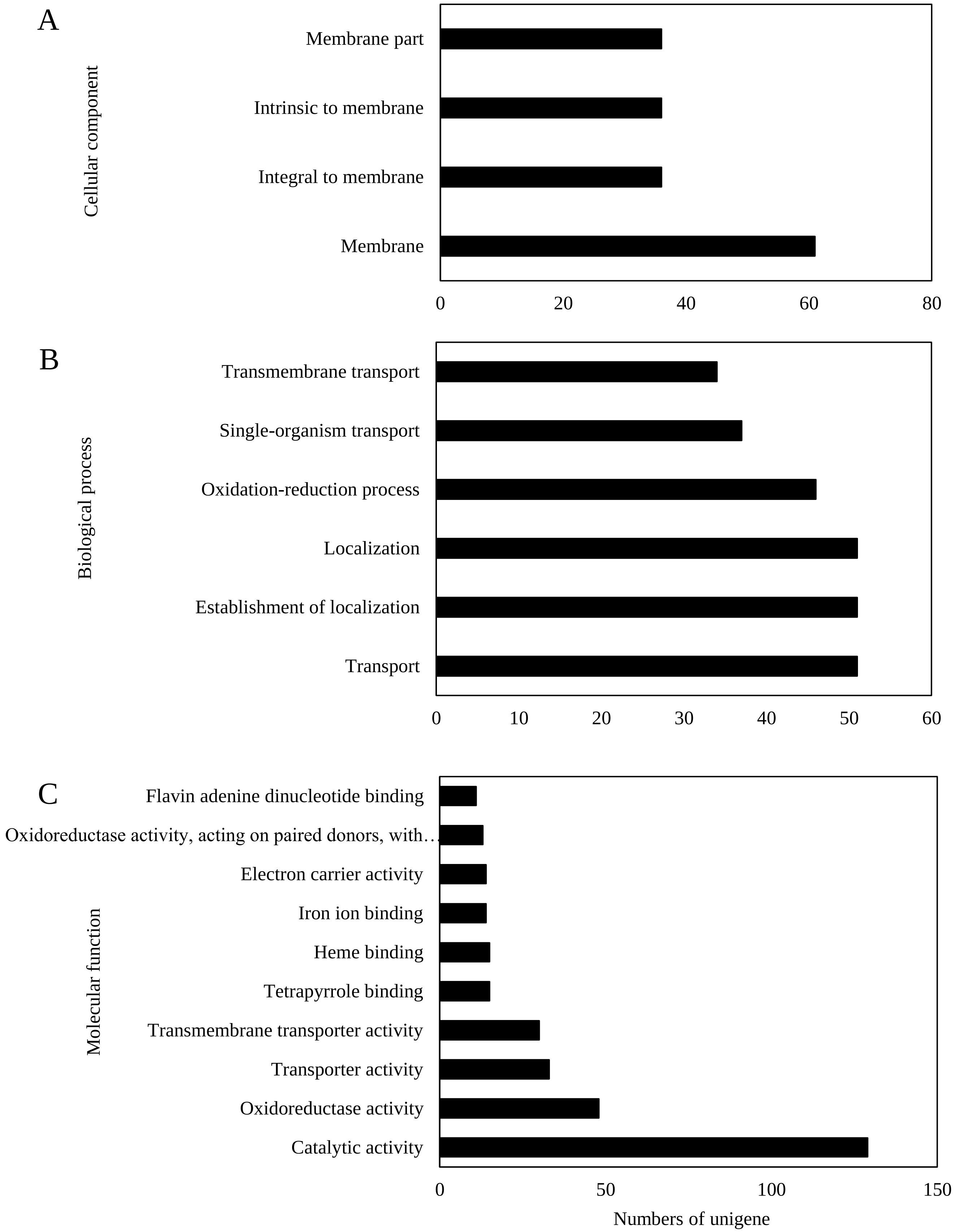

Figure S8. The cellular component (CC), biological process (BP) and molecular function (MF) of gene ontology (GO) categories of the DEGs in *Shiraia* sp. S9 under SNP treatment at 0.02 mM.
